# A sweet deal for domestic industry: the political economy and framing of Vanuatu’s sugar-sweetened beverage tax

**DOI:** 10.1136/bmjgh-2023-012025

**Published:** 2023-10-09

**Authors:** Lana M Elliott, Gade D Waqa, Sarah L Dalglish, Stephanie M Topp

**Affiliations:** 1 College of Public Health, Medical and Veterinary Sciences, James Cook University, Townsville, Queensland, Australia; 2 School of Public Health and Social Work, Queensland University of Technology, Brisbane, Queensland, Australia; 3 Pacific Research Centre for the Prevention of Obesity and Non-Communicable Diseases (C-POND), Fiji Institute of Pacific Health Research, Fiji National University, Suva, Fiji; 4 Bloomberg School of Public Health, Johns Hopkins University, Baltimore, Maryland, USA; 5 Institute for Global Health, University College London, London, UK; 6 Nossal Institute for Global Health, University of Melbourne, Melbourne, Victoria, Australia

**Keywords:** health policy, nutrition, prevention strategies, diabetes, qualitative study

## Abstract

**Introduction:**

The Government of Vanuatu introduced an excise tax on sugar-sweetened beverages (SSBs) in 2015. While lauded for its alignment with the WHO’s Best Buys recommendations for addressing non-communicable diseases (NCDs), little is known about the tax’s adoption process or whose interests it serves.

**Methods:**

Using case study methodology, this study examined how and why Vanuatu’s SSB tax was introduced. Policy documents, key informant interviews (n=33) and direct observations were analysed using theories of policy analysis, power analysis and postcolonial theory to map the policy’s adoption, surrounding political economy and the ideas, interests and institutions that shaped the tax and its framing.

**Results:**

The SSB tax emerged during a politically and economically unstable time in Vanuatu’s history. The tax’s links to the national health agenda were tenuous despite its ostensible framing as a way to combat NCDs. Rather, the tax was designed to respond to tightening economic and trade conditions. Spearheaded by several finance-focused bureaucrats, and with limited input from health personnel, the tax targeted less frequently consumed carbonated SSBs (which are mostly imported) without any revenue reinvestments into health. Driven by the desire to generate much-needed government revenue and instal domestic protections via selective implementation and carve-outs for local producers, the Vanuatu SSB tax did meet national objectives, just not the dual health and economic ‘win-win’ projected by the NCD Best Buys.

**Conclusion:**

Vanuatu’s SSB tax adoption process reveals the limitations of decontextualised policy recommendations, such as the NCD Best Buys, whose framing may be overcome by local political realities. This research highlights the need for further political economy considerations in global health recommendations, since contextual forces and power dynamics are key to shaping both how and why policies are enacted and also whose interest they serve.

**WHAT IS ALREADY KNOWN ON THIS TOPIC**

- Health taxes, including those on sugar-sweetened beverages (SSBs), are increasingly being used by countries to respond to escalating levels of obesity and non-communicable diseases (NCDs).
- SSB taxes are promoted as part of the WHO ‘NCD Best Buy’ package of cost-effective interventions.

**WHAT THIS STUDY ADDS**

- In Vanuatu, the SSB tax’s introduction was driven by trade and economic motives despite being publicly framed as a health initiative, likely limiting its positive impact on population health.
- The policy process and outcome of the SSB tax were shaped by contextually specific power dynamics related to domestic fiscal pressures and trade liberalisation, which decontextualised policy recommendations such as the NCD Best Buys are often ill-equipped to capture.

**HOW THIS STUDY MIGHT AFFECT RESEARCH, PRACTICE OR POLICY**

- The effectiveness of policies in promoting improved population health rests as much with factoring in unique political economy forces as it does with aligning key technical recommendations.

## Introduction

In response to the global rise in non-communicable diseases (NCDs), the WHO has recommended a suite of policies called the NCD ‘Best Buys’.^1–3^ Encompassing measures deemed cost-effective and framed as a health and economic ‘win-win’, this policy package was originally introduced in 2010 and includes taxes on sugar-sweetened beverages (SSBs).^1 2 4–6^ Evidence suggests that well-designed and targeted taxes on SSBs can be effective in limiting their consumption,^7 8^ positively contributing to efforts to curb obesity and NCDs while concurrently reducing health expenditure and encouraging health system reinvestments.^9–12^ However, despite increasing uptake of the Best Buys, including SSB taxes in 45 countries,^13^ there remains limited understanding of how these policy recommendations interact with complex local political economies.

The Pacific Island nation of Vanuatu introduced a 50vt (US$0.41) per litre excise tax on SSBs in 2015. With limited prior evidence of addressing the increasingly unhealthy food environment in-country,^14 15^ this measure followed regional leaders’ declaration of NCDs as ‘a human, social and economic crisis’ and call for multisectoral national and regional responses.^16^ The first SSB tax in the region was adopted in Nauru in the 1980s.^17^ Targeted taxes on calorie-dense nutrient-poor discretionary foods and beverages are identified in the Pacific NCD Roadmap as ‘a strategically important option’ for addressing NCDs.^18^ As such, Vanuatu’s SSB tax has been lauded by WHO and other health bodies as proactive uptake of the Best Buys recommendation.^3 19^ Yet the historic use of SSB taxes in the Pacific predates the escalation of NCDs, and SSB taxes themselves sit at the intersection of health, trade, economic and private sector interests. This raises important questions regarding what truly catalysed the design and enactment of Vanuatu’s SSB tax, and with what implications.

### Study context

The small island state of Vanuatu is an archipelago, with a population of 300 000 spread across 83 islands and 660 000 km^2^ of Pacific Ocean. A previous colony of the French and British, Vanuatu gained independence in 1980. Contemporary Vanuatu thus combines vibrant heterogeneous Indigenous sociocultural structures and languages with a legacy of cobbled-together colonial legal and political structures.^20 21^

The nation’s Westminster system of government comprised a 52-member unicameral parliament. Coalition-based political leadership is the norm with major parties lacking sufficient numbers to govern independently.^22^ However, with fluid coalitions strongly influenced by patronage, votes of no confidence and unstable leadership are common.^23 24^ A chiefly system, the Malvatumauri Council of Chiefs, is enshrined in the Vanuatu Constitution,^25^ and holds considerable power in influencing public and political agendas on socio-cultural issues.^23^

Exploitation of people and resources by colonial powers has had intergenerational impacts on Vanuatu’s gross domestic product. Vanuatu’s continued status as an offshore finance centre or ‘tax haven’, originally orchestrated to serve the interests of colonial authorities and money markets, has been used to attract investors but concurrently constrains national revenue generation.^26 27^ In addition, Vanuatu is ranked among the world’s most disaster-prone countries, and frequent natural disasters are costly to the economy, state building and human health.^28^ These combined forces undermine national development and have meant Vanuatu only recently graduated from ‘Least Developed Country’ status in 2020.^29–31^

Vanuatu is home to two major SSB suppliers: Vanuatu *Brewing* Limited and Vanuatu *Beverage* Limited.^32^ Established in 1989, Vanuatu Brewing, colloquially (and hereafter) referred to as ‘Tusker’ after its beer label, is the nation’s largest *importer* of SSBs and holds the exclusive wholesale license for Coca-Cola products.^33^ This exclusive license ensures Tusker a domestic competitive advantage in importing SSBs at a lower cost than its competitors.^30 33^ The second major SSB supplier, Vanuatu Beverage, was established pre-Independence in 1972 and produces SSBs locally, including the popular domestic brand ‘Splashe’.^34^ Other retailers can directly import or produce SSBs, however Tusker-imported Coca-Cola and Vanuatu Beverage’s product Splashe, represent the major market share.^35–37^

In 2012, Vanuatu was importing nearly two million litres of SSBs annually^38^ much of it due to increases in the importation of Fijian-produced SSBs between 2006 and 2012, under the Melanesian Spearhead Group (MSG) Free Trade Agreement. That agreement dismantled import tariffs between signatory Pacific Island countries, increased imports of tariff-exempt goods including SSBs^38^ and was more generally illustrative of the trade and economic imperatives that accompanied Vanuatu’s path towards trade liberalisation in the late 2000s and early 2010s. Accession to the World Trade Organisation (WTO) and the negotiation of multiple regional trade agreements, for example, significantly cut tariff collection on imported goods.^39^

At first glance, therefore, the introduction of an SSB tax in 2015 appeared inconsistent with Vanuatu’s otherwise industry-friendly economic stance and was framed as a significant win by pro-health champions in the bureaucracy.^40 41^ Yet the tax emerged at a politically and economically unstable moment in Vanuatu’s history and the rationale for enactment and technical policy detail are more complex than they initially appeared.^42 43^ Using political economy analysis, this study seeks to unpack the ideas, interests and institutions that shaped the Vanuatu SSB tax.

## Methods

### Theoretical positioning and methodology

This research used case study design to examine how and why the Vanuatu SSB tax was adopted. Case study design is commonly used in policy analysis given the integral role context plays in shaping policy.^44 45^ The study was grounded in critical realism, which centres the interrogation of power and its role in shaping interactions between people and processes.^46 47^ Within this ontological positioning, Gidden’s theory of structuration and Bourdieu’s forms of capital were incorporated into the study’s theoretical basis to consider power-related themes.^48 49^ Structuration recognises structure and agency as interdependent; positing that individual autonomy is influenced by, and itself reproduces, embeds and over time may alter, social and cultural structures.^50^ Bourdieu’s forms of capital is focused on understanding sources of power and is a useful scaffold for exploring the embodiment and accumulation of characteristics or conditions that afford power different types of actors in a given setting.^49^ To explore the power dynamics that influenced the formation and enacting of the Vanuatu SSB tax, these theories for exploring the sources and manifestations of power were used in conjunction with the ideas, interests and institutions framework.^51–53^ This framework provides a heuristic for considering the inter-relationship between knowledge and information (ideas), diverse agendas held by actors or groups (interests) and the sociopolitical context which remains governed by particular rules and norms (institutions).^51–53^ Postcolonial theory was also central in considering the origins and impacts of widely held global health norms and served as an central reflexive anchor for the study’s non-Ni-Vanuatu authorship team (see online supplemental file 1 for authors’ reflexivity statement).^54 55^ This theoretical positioning and methodology allowed for exploration of the policy’s surrounding political economy; focusing on the complex ideas, interests and institutions at play.^56^

10.1136/bmjgh-2023-012025.supp1 Supplementary data

### Data collection and management

Data were collected from January 2020 to April 2021. Using a multimethod qualitative approach, this study combined key informant interviews, document analysis and direct observations collected and analysed iteratively. This approach allowed for the collation of diverse perspectives and interpretations of events surrounding the introduction of the SSB tax; permitting a multidimensional analysis capable of producing rich insights and enhanced credibility of findings.^57 58^

A total of 33 key informant interviews were conducted using purposive and snowball sampling. Informants represented diverse sectors nationally and regionally and included policymakers, bureaucrats and representatives from industry, media, academia, civil society and development partner organisations (table 1). The interview guide drew on Walt and Gilson’s policy triangle and used semistructured open-ended questions,^59^ permitting informants to reflect on their own knowledge and experience. Interviews lasted 1 hour on average and were facilitated in English or Bislama by LME who is a native English speaker and fluent in Bislama. Ten interviews were conducted in person in February 2020. The remaining interviews (n=23) were conducted via Zoom or phone due to COVID-19 travel restrictions. All interviews were audio recorded with participants’ consent and transcribed verbatim. A total of 157 archival records and documents were also collected for analysis (table 1). Many were provided by informants while others were sourced iteratively through national and regional data repositories, media channels and online searches using terms linked to relevant social, political and health system decisions and events. A broad date-range was set for document inclusion (2000–2020) to ensure that policy-relevant events were historically informed and could be traced forward to understand their implications. Data collection also included 27 unstructured research memos documenting observations related to sociocultural and political events, bureaucratic processes, policy debates as well as non-verbal observations from interviews, interactions and the review of national and regional traditional and social media channels (table 1).

**Table 1:** Number and type of study informants, archival records and observations

Informant type	#	Document type	#	Observation type	#
Government health	5	Government of Vanuatu reports and policy documents	31	Interview/informal interactions observations	11
Government finance	4	Media reports	19	Policy observations	3
Political	1	Press releases, communiqués and committee reports	9	Bureaucratic observations	3
WHO country and regional office	3	Regional reports and policy documents	12	Sociocultural observations	1
World Bank	3	Global reports and policy documents	22	Documentation observations	1
Melanesian Spearhead Group	1	Vanuatu development partner reports	7	Media analyses	8
Secretariat of Pacific Community	5	Vanuatu specific academic papers	23		
Pacific Island Forum Secretariat	1	Regional and global academic papers	15		
Civil society	1	Corporate documents and websites	4		
National media	1	Parliamentary Hansard	3		
SSB industry	1	Acts of parliament	4		
National development partner	2	Meeting minutes and correspondence	8		
Regional academics and commentators	5				
**Totals**	**33**		**157**		**27**

SSB, sugar-sweetened beverage.

### Analysis

All data were entered into NVivo V.12. All data sources were reread, and recordings replayed in an initial data familiarisation phase. The first round of thematic coding was undertaken inductively by LME with transcript data live coded to minimise misinterpretation.^60^ Initial codes were discussed among the authorship team and considered in light of theories of power and policymaking, permitting subsequent coding and refinement. Specific codes for dates and events facilitated process mapping. Further rounds of coding interspersed with member checking and peer debriefing allowed authors to test theories and derive meaning.^61^ Analysis drew on synthesised data to map the policy process and diffusion of policy ideas across time and actors. In-depth stakeholder analysis also centred the deductive application of Bourdieu’s capitals to the key actors and relationships identified through interviews, document analysis and observations.^48 49^ Structuration theory was applied to analysis of policy process data via an exploration of agentic and structural power, aiding in the identification of interests and institutions relevant to the SSB tax.

### Patient and public involvement

While community members were not directly involved in the design of this study, the project was informed by the Vanuatu NCD policy and priorities outlined by the Ministry of Health (MOH) (further detail included in online supplemental file 1). Study results will be shared with study participants and relevant stakeholder in written form and through a series of knowledge translation sessions.

### Ethics

Free and informed consent was sought from informants prior to interview. Participation was voluntary, non-remunerated and consent for interview recording was sought separately from participation consent.

## Results

### The Vanuatu SSB tax

The idea of a SSB tax was proposed by the Ministry of Finance and Economic Management (hereafter Ministry of Finance (MOF)) in late 2012. Its instigator was a foreign economist embedded within the MOF. According to one development partner representative, it was conceived of as a ‘revenue gathering exercise’, a view affirmed by another informant:

[The] characterisation of this [the SSB tax] as a revenue raising initiative is absolutely correct.—Finance representative

With existing SSB taxes at least nominally framed as responding to NCDs in other Pacific nations, and with Vanuatu’s own increasing trade and economic pressures, a coalition of MOH and MOF bureaucrats formed around the proposed excise tax. The SSB tax was viewed by proponents as a reputable mechanism aligning the nation’s health, trade and economic priorities. However, despite WHO being a strong advocate of SSB taxes in general, this early coalition did not include WHO representation.

Over more than a year, the MOH–MOF coalition raised the profile and advocated for the idea of the SSB tax. MOH bureaucrats knew that the measure required MOF support to be endorsed by the parliamentary Council of Ministers (hereafter ‘cabinet’) but wanted a tiered, sugar-content based measure, with a portion of revenue earmarked for health. MOF bureaucrats were opposed to earmarking, considering it counter to good public financial management, and wanted a volumetric rather than nutrient-based tax to ease administration. Yet MOF partners also recognised that the ‘health’ framing of the tax (and the support of several senior doctors who advocated for the tax’s putative health benefits)^40^ shielded the proposed measure from being perceived as one designed exclusively to raise revenue at the expense of the domestic beverage industry.

Whenever there’s a local company producing the product … they have a lot of power to talk to the Government, because there’s such a dearth of local industry. For the Government it’s a really important priority that, of those industries that exist, it’s important we listen to them.—Political representative

Thus, the SSB tax was framed as a health initiative by the MOH Senior Finance Officer at a government-wide revenue generation conference in early 2013. In exchange, the MOF-drafted cabinet paper included loose wording around future health investments, although MOF bureaucrats involved said they never intended to pursue earmarking post the tax’s enactment:

From a Ministry of Finance perspective… you would earmark to get public support for the idea of revenue raising. Well, if there’s no need to get public support, no need to earmark I guess.—Finance representative

The cabinet paper was a MOF initiative with limited input from internal or external health stakeholders, including the MOH and WHO. The MOF’s early interest in revenue raising over health outcomes is illustrated by the paper’s recommendation that the tax be volumetric rather than nutrient-based; in other words, based on a standard per litre charge rather than proportional to the beverage’s sugar content. This pragmatic recommendation was based on the relative ease of enforcing volumetric measures, however it provided no incentive for healthier product reformulation. A second recommendation in the paper was that the tax be specifically assigned to *carbonated,* but not to *non*-carbonated SSBs such as the domestically produced Splashe. Non-carbonated SSBs are more widely consumed in Vanuatu,^62^ and domestic industry are powerful local stakeholders (see further below). Only applying the tax to (largely imported) carbonated SSBs thus reduced domestic opposition, but simultaneously limited the tax’s potential health impact.

Furthermore, as a MOF initiative with few health experts involved, fact checking of health-related claims in the cabinet paper was limited, resulting in the inclusion of data from three important but *incorrect* technical sources. First, the cabinet paper assumed that SSB consumption in Vanuatu (and associated health conditions and healthcare costs) would all increase with economic development.^38^ However, the baseline measure of diabetes prevalence at 21% was incorrect. Taken from the NCD STEPS report, this figure was later demonstrated to have resulted from a diagnostic equipment error that inflated diabetes prevalence.^63 64^ Vanuatu’s true diabetes prevalence was 9.3%.^15^ A second incorrect technical source was the Global School Health Survey data which reported student SSB consumption.^65^ The survey data was based on students’ description of their SSB consumption in the previous 30 days, but a regional report mistakenly reported those figures as *daily* consumption, potentially amplifying youth consumption rates 30-fold.^65^ A third technical source was the MOF-led analysis of SSB imports, which were reported to have increased 191% between 2006 and 2012, yet were not contextualised with reference to the concurrent 255% increase in tourism (tourists are key SSB consumers) over the same period.^38 66^

Due in part to the urgency conveyed by these (incorrect) technical sources, the tax gained strong bureaucratic support throughout 2013 and into 2014 despite an unstable political climate, surviving two changes in governments, two different health and three different finance ministers. As bureaucrats refined their pitch to incoming ministers, the proposed tax rate increased from 30vt (US$0.26) to 50vt (US$0.41), an adjustment made to increase the appeal of the tax among successive revenue-concerned governments, rather than based on price elasticity or health impact concerns.

In the end, it went up simply because it didn’t raise enough revenue… 30vt doesn’t make it attractive enough. Where 50vt you could almost, perhaps, maybe, get 100 million vatu from this policy. Which sounded like a nice, round, big number.—Finance representative

**Figure 1:**
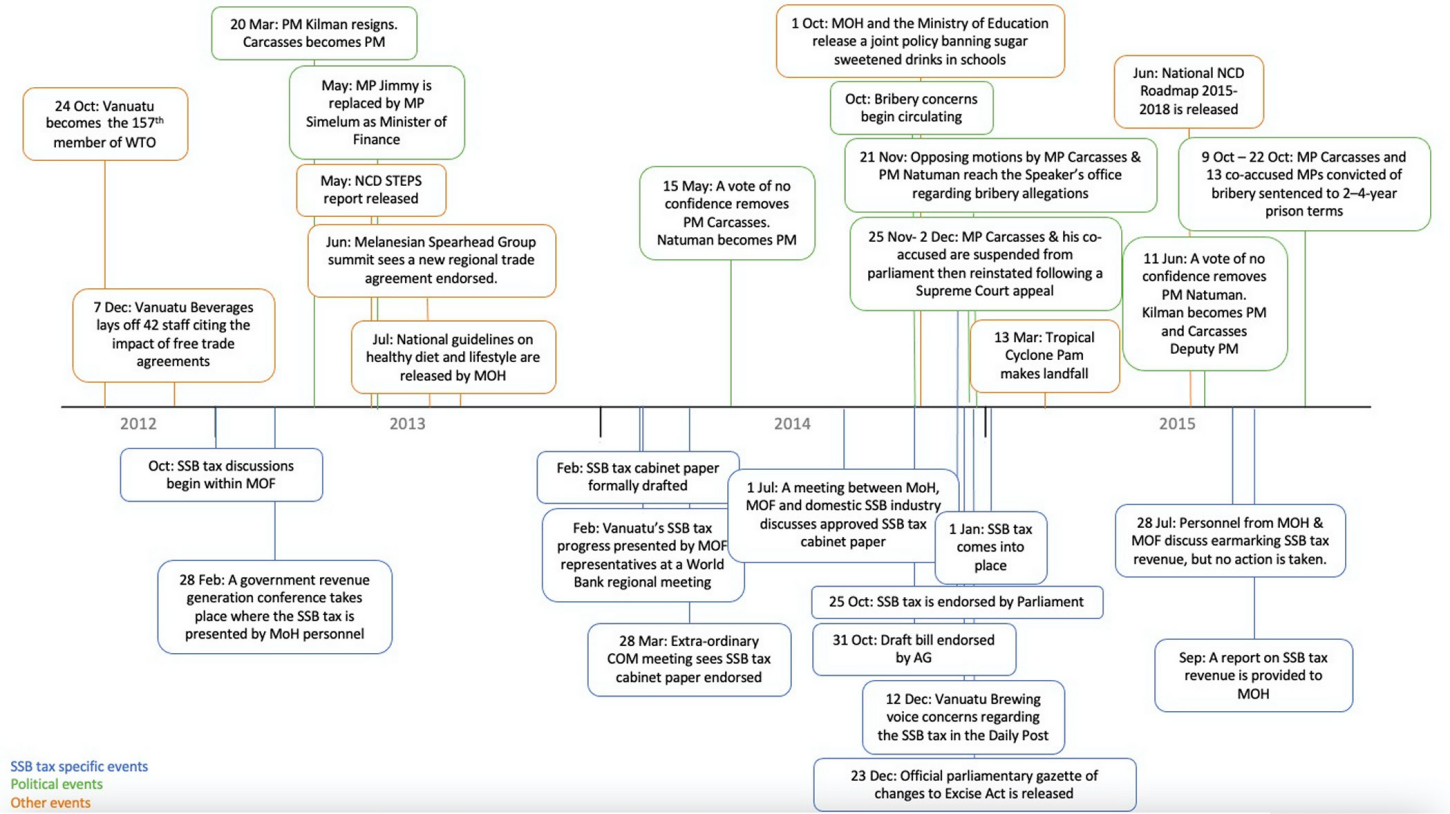
Timeline of events surrounding the Vanuatu SSB tax. MOH, Ministry of Health; NCD, non-communicable disease; SSB, sugar-sweetened beverage.

The process of securing ministerial support for the tax spanned 18 months, with the cabinet paper finally endorsed by cabinet in March 2014 (figure 1). Less than 6 weeks after that endorsement, a vote of no confidence in Prime Minister (PM) Carcassess saw another change in government. It then took until October 2014 for the draft bill to be endorsed by the Attorney General and, more than 2 years from its initial proposal for the finalised bill to reach parliament in November 2014. Despite some objections, the motion passed by a slim majority, bringing the SSB excise tax into force as of 1 January 2015. However, news of the SSB tax was subsumed by the parliamentary motion that directly followed: a case of widespread political corruption that would upend government and embroil 16 then-sitting MPs (30% of parliament), captivating public and media attention that day and in the months that followed.^24 42 43^ Three months later, Vanuatu’s situation would shift permanently when a category five cyclone, Cyclone Pam, decimated the country in one of the worst natural disasters in its history.

### The SSB tax political economy

#### The need for government revenue

Vanuatu’s fiscal situation and the need to raise revenue were pivotal drivers of the SSB tax. As Vanuatu moved to graduate from the United Nations ‘Least Developed Country’ status, preferential treatment by the aid and trade communities alike was projected to diminish.^67^ The slowing in aid following the global financial crisis had also forced the Vanuatu Government to consider its future economic independence.^68 69^ The need for government revenue was also sharpened by a global and regional trade climate characterised by trade liberalisation and new trade arrangements.^70 71^ For Vanuatu, this included WTO accession, full implementation and extension of the MSG free trade agreement and early negotiations of the Pacific Agreement on Closer Economic Cooperation (PACER plus) deal.^39^ Key motives for engaging in trade deals included regional solidarity, Vanuatu’s growing recognition as a regional player and the promise of economic payoffs associated with greater access to export markets. While trade commitments raised Vanuatu’s profile as a trading partner, the concurrent removal of import tariffs and other barriers to trade increased competition for domestic producers and placed additional strain on the net importing nation’s fiscal reserves.

I’d argue that this whole period there’s always been this focus on government revenues.—Health representative

Vanuatu over time had signed free trade agreements with the Pacific Islands and a whole bunch of others and ascended to WTO. So the tax base on the import duty side was slowly eroding. And there was a feeling within government that they needed to find a way to raise revenue.—Finance representative

Uncertainty regarding foreign aid and reduced import revenue forced the Vanuatu Government to look for internal mechanisms to increase funds. Yet having traditionally encouraged foreign investments and business interests through a low tax base,^26^ there was powerful opposition among the nation’s business community to introducing an income tax.^27^

The government in February 2013 wanted to find a way to raise revenue but not implement an income tax… They had a big conference at Le Legon, and brought all of the ministries in, and every single ministry presented on an idea [to raise revenue].—Finance representative

It was in this context that the proposal for the SSB tax emerged, framed by the MOH–MOF coalition as responding to the nation’s growing NCD burden but more critically, addressing the increasing need for revenue. Informants also noted that the excise tax on SSBs was characteristic of the response of governments in many import-reliant economies to trade liberalisation; that is, switching from import-tax to taxation through excise provisions. Import taxes—those that apply to imported and not domestically produced goods—are the target of trade deals and liberalisation. By swapping import taxes for excise tax provision—those that apply to domestic and imported goods—countries can secure ongoing revenue while ensuring compliance with trade deals. Indeed, bureaucrats and politicians at the time of enactment (as reflected in Hansard)^72^ and during later interviews, reflected that the revenue generating potential of the tax was far more important to those within government, than its putative health benefits.

Any kind of vague revenue collection policy that had any sort of reasonable justification was going to stick. And this [the SSB tax justified on health grounds] was one of them.—Finance representative

#### Political and bureaucratic instability

Alongside challenging economic conditions, chronic political instability in the early to mid-2010s shifted relationships and power dynamics, creating an unexpected path to legislation for the SSB tax. The 18 month period stretching from late 2012 to mid-2014 saw three PMs and frequent portfolio reshuffles by coalitions trying to maintain internal support. With frequent parliamentary votes of no confidence and the nation’s largest case of political corruption, politicians’ attention was focused more on day-to-day political power struggles than issues of policy.

There was a great deal of criminality at that time. There was a great deal less focus on governance.—Media representative

Indeed, the level of political instability prompted some interviewees to describe the eventual enactment of the SSB tax as a stroke of sheer luck.

The Minister of Finance through that period changed three or four times, I think. So that’s just complete luck that, you know, he just didn’t go [say] one day: ‘No we’re not doing this’.—Finance representative

On the one hand, ownership of the SSB tax proposal by the MOH–MOF coalition protected the measure from the political machinations by positioning it as politically agnostic. Ongoing bureaucratic support for the tax, despite the surrounding political uncertainty, sustained momentum and, importantly, ensured that the tax’s economic impetus—and health framing—were maintained during its passage through parliament. In fact, the challenging political climate shifted considerable power to the bureaucracy in designing and enacting policies like the SSB tax. Several informants highlighted that throughout Vanuatu’s history, it was during times of political *instability* that policy change was more prolific but could risk insufficient strategy when lacking bureaucratic oversight.

When the government is changing consistently, they’ll pass any old thing through. And they just don’t care. They just care about, you know, who’s the next prime minister.—Finance representative

On the other hand, the unstable political environment negatively impacted many working relationships between ministers and bureaucrats, and rulings by the Public Service Commission saw a high turn-over of key bureaucrats.^73^ These conditions particularly impacted the MOH, with all three directors concurrently suspended under the 2013 Carcasses government.^74^ This instability weakened MOH actors’ role in the SSB tax. With previous directors caught in protracted legal battles for wrongful dismissal,^74 75^ the MOH–MOF SSB tax coalition lost its health champions and those who remained were less vocal. By the time of SSB tax’s implementation, many senior health bureaucrats had not been reappointed, translating into a lack of institutional memory that negatively impacted how health guidance fed into implementation or evaluation.

There’s institutional memory lost about what was done before, that’s definitely true. You have people having to reinvent the wheel a lot because there’s just no memory of these things that’ve already been done.—Political representative

The political (and subsequent natural) disasters saw public and political awareness of the SSB tax dissipate while government personnel changes meant that the MOH–MOF SSB tax coalition were unavailable to guide future steps. The absence of this vanguard left the policy’s implementation vulnerable, a weakness that was soon exploited by the domestic SSB industry.

It was passed but then immediately the lobbying began… In the end … they got a lot of exemptions for other things like import duties et cetera. At the end of the day, [the SSB tax] didn't make any difference.—Finance representative

#### The power of the domestic industry

The power of the domestic industry was visible in the differential treatment of the domestic and foreign SSB industries in the passage of Vanuatu’s SSB tax. According to interviews, the interests of the foreign SSB industry did not factor into bureaucratic or political thinking around policy design and advocacy. Rather, the socially and politically protected status of the domestic SSB industry, combined with a stretched bureaucracy, resulted in domestic carve-outs and concessions both before and after enactment.

Prior to enactment, for example, it was recommended that the tax be assigned to tariff item 22.02 (encompassing *carbonated* beverages). This tariff item did not include locally produced and more widely consumed *non*-carbonated beverages (including Splashe) which were instead assigned against tariff item 20.09 that attracted zero excise tax.

I said to [the foreign economist], I think you’re going to miss Splashe. And it’s basically the one causing diabetes in my opinion. Almost single-handedly the cause of diabetes, that bloody product. So I was like, it’s not carbonated so what are you going to do?—Finance representative

Such concessions were achieved in the context of increasing trade liberalisation and a slowing national economy, which heightened concerns regarding local industries’ viability, encouraging the powerful domestic SSB industry to demand further government protections before and after the tax’s enactment.^76 77^ While domestic beverage producers, notably Tusker, were initially vocal in their opposition to the tax,^36^ as the policy coalition solidified, their lobbying was said to have shifted behind closed doors; influencing *how* the tax was designed rather lobbying for its scrapping altogether. One informant described industry lobbying as focused on shifts in the language used during this phase:

Yeah, maybe that’s where their lobbying focussed on, changing the wording. —Health representative

Records of parliamentary debate also demonstrate an explicit focus on how the tax could be used to carve out domestic protections that had been eroded through trade liberalisation.

The Hon. Minister Simelum thanked the Hon. Chabod for his comments concerning the protection of local industries, and highlighted the financial supports granted to ‘Tusker’, and welcomed further queries from the company. He added that the Government was not seeking to protect the company itself but the entire industrial sector.—Parliamentary Hansard^72^

The way the SSB tax was structured and administered thus belied its health framing and highlighted the importance of domestic industry interests.

In contrast, the foreign SSB industry exerted relatively little influence over the process. While regional Coca-Cola representatives appeared disapproving of the tax in media reports,^36^ few informants identified foreign industry actors as having any substantial influence. Some speculated that political instability made it challenging for the foreign industry to identify a receptive audience among politicians. Others suggested that foreign beverage industries saw a strategic advantage in their interests being represented by intermediaries such as the Vanuatu Chamber of Commerce and Industry or domestic actors. Still others suggested that the SSB market in Vanuatu may have been considered too small to intervene or that multinational corporations suspected that the tax would not impact sales. Local industry actors also noted that exclusive supplier contracts between multinational suppliers and local distributors were poorly regulated in Vanuatu. This lax regulation spread the tax’s impact across the market and fuelled local competition, potentially preventing a united opposition to the tax from forming.

## Discussion

Improved population health was far from the driving force behind Vanuatu’s SSB tax idea. Instead, the SSB tax was introduced as a revenue-generating measure in response to increasing trade liberalisation and domestic fiscal pressures. Political instability shifted political and bureaucratic power dynamics, making it difficult to sustain a health focus within the MOH–MOF coalition and incentivising a focus on immediate domestic fiscal interests rather than longer-term strategic goals such as population health. These combined circumstances granted considerable power to the domestic SSB industry to lobby for carve-outs and additional concessions, severely limiting the potential health impact of the tax.

In the case of Vanuatu, how the SSB tax was envisaged, designed and implemented thus belies the optimistic health framing that often accompanies such measures. Our findings reveal that assumptions around policy motives, potential opposition and the ‘win-win’ outcomes inherent in decontextualised policy recommendations, such as WHO’s NCD Best Buys, may be severely tested by real-world circumstances and the interplay of contextually specific interests and institutions.

Global policy recommendations, such as the NCD Best Buys, are often built on the premise that improved population health is a normative good pursued by those in power. Indeed, central to the universal recommendations of NCD Best Buys by the WHO is the idea that improved population health is the primary, if not only, rationale for SSB taxes. Yet health goals frequently conflict with the interests of economically and politically powerful actors, particularly in the case of NCDs.^78–80^ As findings from this study demonstrate, the assumption that health is foremost in political decision-making fails to account for: (1) the fact that prevailing political norms may mean health-interested actors do not always hold great power in political decision making; and (2) that those with power to shape political agendas may have less or no interest in population health as an outcome (particularly given the diffuse electoral benefit of improved population health compared with the proximate and concentrated benefits of supporting industry interests).^81 82^ Examination of Vanuatu’s handling of SSB taxes (as well as tobacco, alcohol and marijuana)^83–85^ reveals how ideas that shaped the SSB tax policy design were determined at the intersection of stakeholder interests and the specific combination of formal and informal rules that influence the dynamics of Vanuatu’s political scene,^49^ such that *health* actors’ influence remained significantly limited unless, and even when aligned with, the interests of more powerful government or private sector actors.

Policy recommendations focusing on content or ideas alone do not always square with the reality that political trade-offs are an inherent part of the policymaking process and policy outcomes. As argued by Whyle and Olivier, ‘values influence policy-makers and shape policy making processes’, through their expression in stakeholder interests as well as their role in formal and informal rules that influence how policy goals are both devised and achieved.^86^ Yet to date, considerable policy analysis surrounding WHO’s NCD Best Buys has focused on technical policy content,^2–4 87^ with a relative dearth of literature outlining how the complex policy processes and associated institutions should or could be traversed. Analysis of the policy content alone in the Vanuatu SSB tax case would reveal little about the complex interplay of interests and institutions that ultimately redefined the policy goal. Policy reform is inherently political because it determines who can access valued social goods.^88^ It is hence in the analysis of policy *process* that bidirectional relationship between structural power (ie, what is socially valued) and agentic power (ie, who influences decisions)^48^ is most evident and the ramifications on policy goals and outcomes most apparent. While a longstanding recommendation^59^ to track and genuinely assess the achievement of complex policy goals, policymakers and researchers alike must extend their focus beyond content analysis to also consider policy process and the interests and institutions that shape it, within this domain.

Current SSB tax knowledge has focused on building an investment case,^5 6 87^ showcasing uptake and technical lessons^19 89 90^ and assessing impact in various ways.^7 8 12 91^ However, much less is known about the political mechanisms underpinning SSB taxes or the implications of such on their use or efficacy as a health-promoting measure. Existing research on the political economy of SSB taxes shows a diverse mix of ideas, interests and institutions affecting policy outcomes.^92–94^ However, to complement growing technical know-how and increasing global uptake of SSB taxes, policymakers must also demand greater insights into contextually nuanced political realities and their policymaking implications to ensure that technically valid policies are matched by an in-depth understanding of and ability to navigate the politics at play.

Substantial literature outlines challenges multinational corporations pose to health taxes.^95–97^ The Vanuatu experience adds richness to this literature by revealing less frequently examined corporate forces. In this case, more economically powerful players (large multinational corporations) were not more politically powerful. The domestic SSB industry’s interests were given priority over those of large multinational actors and were a driving force behind the tax. Domestic industry stakeholders’ power to influence the policy process derived from their proximity to decision makers and (cf Bourdieu)^49^ sociopolitical capital and know-how.^49^ Analyses from Bermuda and Colombia have also demonstrated how local industries leverage their sociopolitical knowledge and networks in resisting taxes.^98 99^ Yet this apparent win for domestic industry actors came at the expense of potential health improvements in Vanuatu; a compromise revealing of some of the diffuse and perverse ramifications of trade liberalisation on more import-dependent nations. Further, it highlights the imbalance of global economic power which continues to shape international relations and, by extension, the decisions made by nation-states.

Increasing evidence, buttressed by this study, is challenging the presumption that the mere presence of a tax represents a health win over industry, or indeed any ‘win’ for health. Commercial forces that shape food environments, alongside neoliberal pressures to pursue economic growth in the name of state building, shape sociopolitical institutions and can curtail regulatory measures aimed at protecting health.^31 100 101^ The economic basis underpinning the ‘win-win’ logic of the NCD Best Buys’ recognises the continued importance governments place on economic generation. However, this logic falls short in recognising how these same neoliberal forces can concurrently undermine population health; a fundamental concern that should not be overlooked by health policymakers. Market-driven approaches to development alter where power lies, how agendas are set and whom policies benefit. Assumptions that the presence of an SSB tax with economic benefits equate to a ‘win’ for health should thus be approached with caution.

This study provides comprehensive analysis of the Vanuatu SSB tax passage and is one of relatively few political economy analyses of such types of taxes. It has several strengths and weaknesses. The single case in-depth analysis produces a rich picture of events and decisions influencing the tax, but the specificity of Vanuatu’s circumstances may limit findings’ transferability. The political nature of the tax and the time since its adoption create a risk of misinterpretation of events by interviewees or the research team. We have minimised this risk by triangulating across data sources and iterative analysis. While the authorship team does not include a Ni-Vanuatu perspective, it does include varying degrees of ‘insiders’ and ‘outsiders’ to SSB taxes and the Vanuatu policy context.

## Conclusion

While Vanuatu’s SSB tax was adopted to address government priorities, the nation’s rising rate of NCDs was far from the central concern. Rather, the central objectives were a desire to generate much needed government revenue and instil protections for domestic producers. This case thus reveals several limitations of decontextualised global policy recommendations by highlighting unsupported assumptions about the primacy of health in motivating policy change, the power of health actors compared with other domestic priorities and the compatibility of improving both economic and health outcomes in the context of neoliberalism. This research adds to the evidence that political circumstances are at least as important as technical detail in formulating and implementing sound health-promoting policies. Global policy recommendations have no guarantee of effectiveness without due consideration of sociocultural and political conditions that are necessarily unique to each policymaking context.

## Data Availability

Data are available upon reasonable request.
