# Supplementary material for: A sweet deal for domestic industry: the political economy and framing of Vanuatu’s sugar-sweetened beverage tax
Source: BMJ Glob Health. 2023 Oct 9;8(Suppl 8):e012025. doi: 10.1136/bmjgh-2023-012025 (PMC10565185; doi:10.1136/bmjgh-2023-012025)
Supplement: Supplementary data [file bmjgh-2023-012025supp001.pdf]

Domain	Guiding Question	Authors' Response
Study conceptualisation	1. How does this study address local research and policy priorities?	This study was initially conceived by LME while writing the Vanuatu NCD Policy and Strategic Plan 2016-2020. As such, the study directly links to strategic area 2.1.4 of the 2016-2020 plan and the current NCD Policy and Strategic Plan 2021 – 2030. Its focus on understanding dynamics underpinning the health sector's use of tax measures to improve population health also links with priorities outlined in the National Roadmap for Non-Communicable Diseases and the regional equivalent, Non-Communicable Disease Roadmap Report. Further, this study directly responds to calls for more research into how tax measures have been used in Vanuatu, outlined in a Ministry of Health 2016 report titled 'Fiscal Measures for the Prevention and Control of NCDs in Vanuatu', which was co-authored by LME when working with the Government of Vanuatu.
	2. How were local researchers involved in study design?	Ni-Vanuatu colleagues were not directly involved in the study's design. The study was conceptualised by LME who has spent 9 years working to strengthen health policymaking in Vanuatu. This research forms part of LME's PhD studies under the supervision of ST, SD and Pacific researcher, GW.<br>The study's design was endorsed by the Director of Public Health and the Vanuatu Ministry of Health Executive as part of the Human Research Ethics Committee approval process.
Research management	3. How has funding been used to support the local research team(s)?	Local research teams were not involved in the study.
Data acquisition and analysis	4. How are research staff who conducted data collection acknowledged?	All data were collected by LME.
	5. How have members of the research partnership been provided with access to study data?	All members of the research team have access to the original data files and NVivo collated project file.
	6. How were data used to develop analytical skills within the partnership?	LME engaged in frequent discussions with the authorship team during the analysis phase of the study to build analytical research skills and test hypotheses. Additional analytic input was provided by all authors during the study's write up and refinement phases contributing to further advance the clarity of the study's findings and provide additional learning opportunities.
Data interpretation	7. How have research partners collaborated in interpreting study data?	All authors contributed to the iterative interpretation of results during the analysis and write-up phases of the study
Drafting & revising for intellectual content	8. How were research partners supported to develop writing skills?	LME, as a PhD student and early career researcher, was supported in developing and refining academic writing skills by the ST, SD and GW as senior academics.
	9. How will research products be shared to address local needs?	This study will be published as open access. A series of knowledge transmission sessions have also been embedded within the study design of the broader thesis to ensure key findings are provided to Ni-Vanuatu and regional decision-maker in both oral and written form.
Authorship	10. How is the leadership, contribution and ownership of this work by LMIC researchers recognised within the authorship?	The design of and finding from this study have benefited greatly from input provided by Fijian-based Pacific academic and co-author GW. As a regional leader in NCD research, GW's technical insights and mentorship have aided in ensuring the integrity of research findings.
	11. How have early career researchers across the partnership been included within the authorship team?	LME is the study's first author and is both a PhD student and early career researcher.
	12. How has gender balance been addressed within the authorship?	All members of the research team are female.
Training	13. How has the project contributed to training of LMIC researchers?	The training of LMIC researcher was not a core focus of this research however collaboration and ongoing discussions between the authorship team provided learning opportunities for LMIC and HIC-based authors. Knowledge transmission sessions embedded in the design of the broader thesis to which this study is a part will also, where appropriate, extend to supporting Ni-Vanuatu colleagues to advance their own qualitative research skills and ability to translate research findings into action.

Infrastructure	14. How has the project contributed to improvements in local infrastructure?	This project has not directly contributed to the improvement of local infrastructure.
Governance	15. What safeguarding procedures were used to protect local study participants and researchers?	Human research ethics approval was granted for this project by the Vanuatu Human Research Ethics Committee and the James Cook Human Research Ethics Committee.<br>Particular safeguards to ensure the safety and anonymity of key informants included the availability of study information and consent forms in both English and Bislama, the inclusion of an English-Bislama bilingual interviewer (LME), the de-identification of research informants in data analysis, write up and publication. Consent to record interviews was also collected separately to participation consent and consent document was stored separately to interview data.<br>Significant changes were also made to the data collection method (i.e. shifting to remote interviews) to protect the health of the research team and study informants following the outbreak of COVID-19.
